# Changing the Mandibular Position in Rowing: A Brief Report of a World-Class Rower

**DOI:** 10.3390/jfmk9030153

**Published:** 2024-08-30

**Authors:** Filipa Cardoso, Ricardo Cardoso, Pedro Fonseca, Manoel Rios, João Paulo Vilas-Boas, João C. Pinho, David B. Pyne, Ricardo J. Fernandes

**Affiliations:** 1 Centre of Research, Education, Innovation and Intervention in Sport (CIFI2D), Faculty of Sport, University of Porto, 4200-450 Porto, Portugal; up201200394@edu.fade.up.pt (R.C.); manoel.rios@hotmail.com (M.R.); jpvb@fade.up.pt (J.P.V.-B.); ricfer@fade.up.pt (R.J.F.); 2 Porto Biomechanics Laboratory (LABIOMEP-UP), Faculty of Sport, University of Porto, 4200-450 Porto, Portugal; pedro.labiomep@fade.up.pt; 3 Faculty of Dental Medicine, University of Porto, 4200-393 Porto, Portugal; pinhojc53@gmail.com; 4 Institute of Science and Innovation in Mechanical and Industrial Engineering (INEGI), Faculty of Engineering, University of Porto, 4200-465 Porto, Portugal; 5 Research Institute for Sport & Exercise, University of Canberra, Canberra 2617, Australia; david.pyne@canberra.edu.au

**Keywords:** occlusal splints, mandibular repositioning, elite sport, oxygen uptake, biomechanics, electromyography

## Abstract

We investigated the acute biophysical responses of changing the mandibular position during a rowing incremental protocol. A World-class 37-year-old male rower performed two 7 × 3 min ergometer rowing trials, once with no intraoral splint (control) and the other with a mandibular forward repositioning splint (splint condition). Ventilatory, kinematics and body electromyography were evaluated and compared between trials (paired samples *t*-test, *p* ≤ 0.05). Under the splint condition, oxygen uptake was lower, particularly at higher exercise intensities (67.3 ± 2.3 vs. 70.9 ± 1.5 mL·kg^−1^·min^−1^), and ventilation increased during specific rowing protocol steps (1st–4th and 6th). Wearing the splint condition led to changes in rowing technique, including a slower rowing frequency ([18–30] vs. [19–32] cycles·min^−1^) and a longer propulsive movement ([1.58–1.52] vs. [1.56–1.50] m) than the control condition. The splint condition also had a faster propulsive phase and a prolonged recovery period than the control condition. The splint reduced peak and mean upper body muscle activation, contrasting with an increase in lower body muscle activity, and generated an energetic benefit by reducing exercise cost and increasing rowing economy compared to the control condition. Changing the mandibular position benefited a World-class rower, supporting the potential of wearing an intraoral splint in high-level sports, particularly in rowing.

## 1. Introduction

Rowing is a sport characterized by both strength and endurance where performance is influenced by factors such as aerobic and anaerobic power, physical strength and rowing technique [1]. Given that rowers must develop several skills and capacities to achieve success, multimodal measurement systems are often required to simultaneously evaluate performance, physiology and mechanics in rowing [2]. Notably, during a prominent rowing competition such as the 2000 m race, a rower predominantly relies on aerobic metabolism (~80%) [3,4,5], underscoring the critical role of aerobic performance in this sport. Given this importance, forwarding the mandibular position using an occlusal splint during rowing may prove beneficial since these intraoral splints have been associated with enhanced gas exchange during exercise [6,7,8]. Indeed, the effectiveness of mandibular advancement in enlarging upper airway dimensions is well-documented in the treatment of sleep apnoea [8], sparking considerable interest in its potential applications in the field of sport.

The mandibular protruding position and its related effects on the upper airway, such as increased airflow and decreased airway resistance, have been debated regarding their potential to enhance aerobic exercise performance [7,8]. Nevertheless, although the ventilatory effects of wearing a mandibular protruding splint during exercise have recently gained attention and garnered debate, research on its impact on physiology and performance remains limited. Investigations into how a mandibular forward repositioning splint may enhance performance have primarily focused on recreational and trained individuals during submaximal and maximal running [6,7,8], with less emphasis on other sports and elite athletes. Therefore, we investigated the acute biophysical effects of using a mandibular forward repositioning splint in a World-class rower during a progressive incremental rowing ergometer test.

## 2. Materials and Methods

### 2.1. Participant

A World-class male rower (2012 and 2016 Olympic Games finalist, 2022 European and 2019 and 2021 World Rowing Champion), aged 37 years, with height of 1.87 m and body mass of 79.9 kg, gave his written informed consent to participate after explanation of the project aims, methods, benefits and risks (unconditional withdrawal was possible at any point). The local University Ethics Committee approved the study according to the Declaration of Helsinki.

### 2.2. Design

The rower performed an incremental intermittent protocol in a rowing ergometer (Concept II model D, fixed, Morrisville, VT, USA) in two sessions 24 h apart, once without the use of an intraoral splint (control) and the other wearing a mandibular forward repositioning splint (splint condition). Ventilatory, kinematic and body-surface electromyography (EMG) variables were recorded throughout. The rower was familiarized with the intraoral splint prior to the corresponding testing session without being informed of its potential biophysical effects.

### 2.3. Methodology

The rower completed a low-intensity 10 min warm-up on the rowing ergometer at a self-selected frequency before each test. The incremental protocol consisted of 7 × 3 min rowing steps, interspersed with 30 s intervals for blood sampling collection [9,10]. The initial workload was set at 180 W and increased to 30 W between consecutive steps. The 7th step power was based on the individual 2000 m rowing ergometer performance [11], and six power increments were subtracted to calculate the 1st step power output.

Breath-by-breath data were continuously collected (K5, Cosmed, Rome, Italy), and 3D kinematic variables were recorded at 100 Hz (Miqus, Qualisys AB, Göteborg, Sweden) between the 60 and 120 s interval within each step. Biceps brachii, posterior deltoid and rectus femoris EMG were assessed constantly (right hemibody) by the Trigno Avanti sensors (Delsys, Natick, MA, USA). Muscle surface preparation and sensor placement followed the SENIAM recommendations [12]. A 5 µL sample of capillary blood for lactate concentrations ([La-]) assessment was taken from the earlobe (Lactate Pro2; Arkay, Inc., Kyoto, Japan) at rest intervals between steps and at the end of the protocol [13].

### 2.4. Data Analysis

Oxygen uptake (VO_2_) and ventilation were averaged and compared every 10 s of the last minute of exercise [14], and kinematics were obtained using Theia Markerless software (v2023.1.0.3161_ P14, Theia Markerless Inc., Kingston, ON, Canada). EMG signals were processed in MATLAB R2023b (The MathWorks Inc., Natick, MA, USA), including a band-pass filtration (25–450 Hz), full-wave rectification and linear envelope calculation with a 2nd-order Butterworth low-pass filter (6 Hz). Peak and mean amplitudes were expressed as a percentage relative to the initial value (defined as the EMG mean value during the 1st step) [11], being the instant of peak amplitude normalized to the duration of the propulsive phase (drive). Kinematic and EMG data were examined across ten consecutive rowing cycles in each step performed [15]. The energy expenditure was computed by adding the net VO_2_ values and converting net [La-] into oxygen equivalents [5,13]. The energy cost was determined as the slope of a regression line between energy expenditure and the corresponding power [10], and rowing economy was calculated as the ratio between the power generated and the energy expenditure for each step [14].

### 2.5. Statistical Analysis

Statistical comparisons between control and splint conditions were performed for physiological and biomechanical variables using the paired samples *t*-test in SPSS (version 28.0.1.0, IBM Corp., Armonk, NY, USA). Bioenergetic data were only presented and interpreted as raw (rather than standardized) differences. Cohen’s d (d) was computed to indicate the magnitude of effects (small ≥ 0.2, medium ≥ 0.5 and large ≥ 0.8). Significance was set to 5%.

## 3. Results

**Figure 1:**
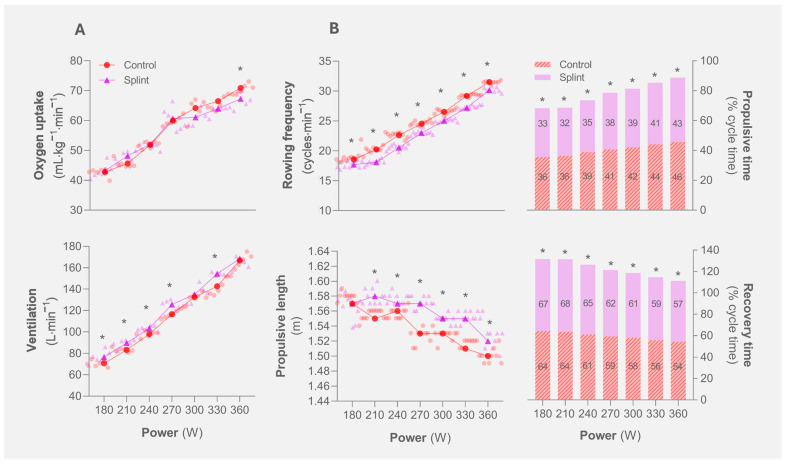
Physiological and kinematic variables (panels (**A**) and (**B**), respectively) assessed during the rowing incremental protocol for the two experimental conditions tested (control vs. splint). * indicates differences between control and splint conditions (*p* ≤ 0.05).

Ventilatory and kinematic variables for both experimental conditions are displayed in Figure 1. VO_2_ values were similar except for the 7th step, where a lower VO_2_ was observed for the splint condition (67.3 ± 2.3 vs. 70.9 ± 1.5 mL·kg^−1^·min^−1^). Moreover, there was a tendency toward a lower VO_2_ at the 5th and 6th steps with the splint compared with the control condition (*p* = 0.08 and 0.06, *d* = −0.89 and −0.99, respectively). The splint condition also exhibited increased ventilation in between the 1st and 4th steps ([76.6–125.6] vs. [70.6–116.4] L·min^−1^) and at the 6th step (154.4 ± 1.5 vs. 142.6 ± 7.8 L·min^−1^).

**Figure 2:**
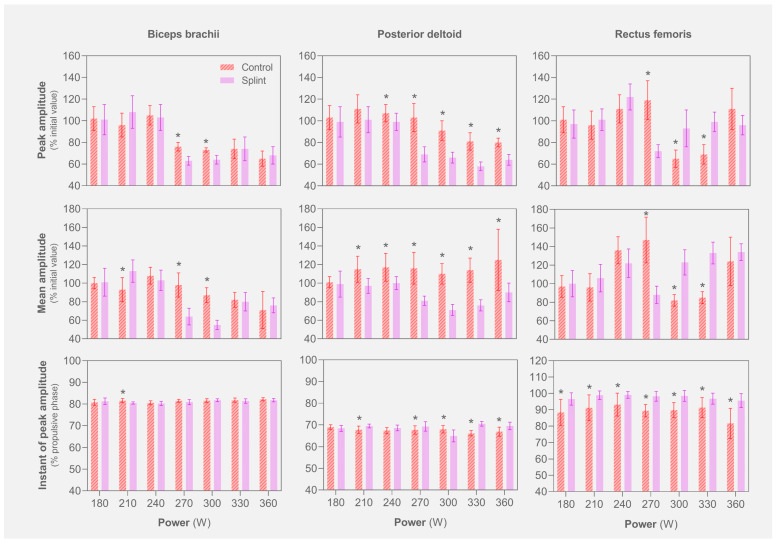
Normalized electromyography for biceps brachii, posterior deltoid and rectus femoris along the rowing incremental protocol for control and splint conditions. The onset and the end of the rowing propulsive phase were 0 and 100%, respectively. * indicates differences between control and splint conditions (*p* ≤ 0.05).

When compared to the control condition, kinematic analysis revealed that rowing frequency was lower ([18–30] vs. [19–32] cycles·min^−1^) and propulsive length was higher ([1.58–1.56] vs. [1.52–1.50] m) for the splint condition along the protocol. Furthermore, shortened propulsive time and extended recovery phases were observed throughout the protocol with the splint. The splint condition exhibited a lower peak and mean biceps brachii and posterior deltoid activation, contrasting with increased rectus femoris activity (Figure 2). The instant of peak activation for the posterior deltoid (except for the 5th step) and rectus femoris consistently occurred later along the propulsive phase in the splint condition, unlike the biceps brachii, where the respective instant occurred earlier (2nd step).

**Figure 3:**
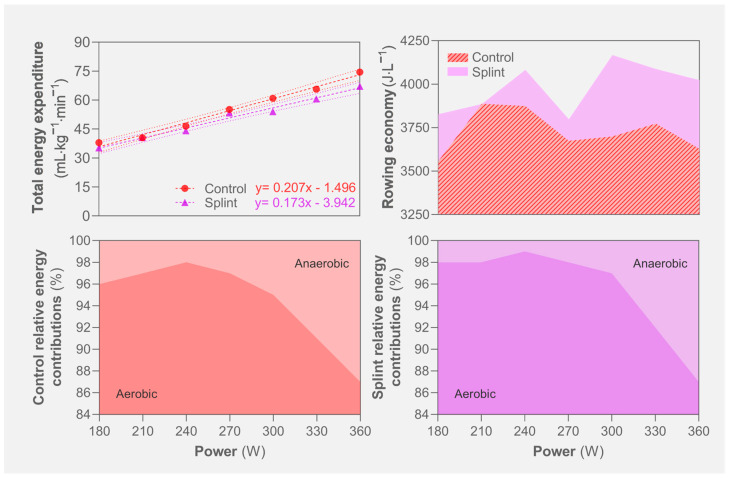
Energetic profile during rowing incremental protocol for control and splint conditions.

The relationships between energy expenditure, rowing economy and the relative energy systems contributions along the protocol are illustrated in Figure 3. A lower slope was observed for the splint condition (0.17 vs. 0.21), indicating a lower exercise energy cost when the mandible was advanced. Moreover, the rowing economy was higher in the splint rather than in the control condition. In both incremental protocol trials, the aerobic and anaerobic energy systems accounted for ~87–99% and 1–13% (respectively) of the energy provided. However, the splint condition showed higher aerobic and lower anaerobic relative contributions than the control condition.

## 4. Discussion

At higher intensities, where respiratory muscles may require ~15% of the whole-body VO_2_, the lower VO_2_ observed by wearing the mandibular forward repositioning splint suggests a reduced requirement to support the respiratory muscle demands compared to the control condition [16]. Moreover, as a significant portion of ventilation occurs orally during exercise, reducing the effort of breathing or mouth airflow resistance with a mandibular forward repositioning splint likely enhances ventilation, as supported by our results. Although conflicting VO_2_ responses are evident while wearing mandibular repositioning splints during exercise [7,8,17], there have been consistent reports of increased ventilation even when different subjects and methodologies were employed [6,8].

Kinematic changes have also been reported while running using splints that modify the mandibular position [6,18]. However, it remains uncertain whether these modifications are a direct consequence or mediated by the ergogenic physiological effects. Our results showed a slower and longer rowing movement, accompanied by a high rowing economy in the splint condition. Indeed, elite rowers may potentially improve performance by slightly lowering their rowing rate (consequently decreasing physiological demands), compensating with increased propulsive force [14]. Given the critical importance of synchronizing breathing with rowing mechanics, the potential reduction in respiratory work and a probable perceived “easier” breathing with the splint might enhance the rowing kinematic pattern.

Rowing is a complex movement that engages multiple muscle groups; however, the activation patterns of the biceps brachii and rectus femoris are frequently investigated when evaluating rowing technique [2]. Although explanations on how intraoral appliances might potentiate strength are inconclusive, certain studies suggest a link between altered occlusal vertical dimension and body muscular activity [19]. These ergogenic strength effects are primarily described for upper body musculature [20,21], with few reports indicating effects on lower limb performance [22,23]. Our data only align with prior research regarding the use of intraoral splints in lower body musculature, although differences in study design and the specific splint tested warrant consideration. Hence, future research should investigate how minor changes resulting from shifts in the lower jaw position may affect muscle activation, potentially leading to a more symmetrical or balanced force production.

The reduced energy cost and enhanced rowing economy noted for the splint condition support an improved rowing efficiency in comparison to the control condition. Furthermore, the observed decrease in anaerobic relative contributions may potentially have delayed the onset of fatigue when the splint was worn. The current ventilatory and energetic findings align with data from a highly trained triathlete who wore an advancement splint during a running incremental protocol [6]. Although the current study is pioneering in demonstrating the effects of mandibular forward repositioning in a high-level performance rower, our findings may not be easily generalized to other athletes or sports, given our small sample size. Additionally, our investigation only assessed the acute response to wearing a mandibular forward repositioning splint during rowing. Therefore, future research should explore the potential chronic effects of using such splints over extended training sessions or an entire competitive season.

## 5. Conclusions

In World-class rowing events, medal rankings are often decided by mere fractions of a second, underscoring the importance of any competitive advantage. Our findings highlight the potential beneficial impact of wearing a mandibular repositioning splint in rowing, a sport where such effects have not been previously explored. Testing a unique subject, as an Olympic finalist and World Champion, highlights the utility of employing a case study design to “open the door” for further investigation into wearing mandibular forward repositioning splints within high-level sport. An altered mandibular position induced by an intraoral splint elicited favourable biophysical and energetic advantages during an incremental rowing test in a Word-class rower.

## Data Availability

All data were contained within the manuscript.
